# Longitudinal Impact of Vision Impairment on Concern About Falling in People With Age-Related Macular Degeneration

**DOI:** 10.1167/tvst.11.1.34

**Published:** 2022-01-25

**Authors:** Ursula E. White, Alex A. Black, Kim Delbaere, Joanne M. Wood

**Affiliations:** 1 Centre for Vision and Eye Research, School of Optometry and Vision Science, Queensland University of Technology, Kelvin Grove, Brisbane, Queensland, Australia; 2 Falls, Balance and Injury Research Centre, Neuroscience Research Australia, Randwick, New South Wales, Australia; 3 School of Public Health and Community Medicine, University of New South Wales, Kensington, New South Wales, Australia

**Keywords:** age-related macular degeneration (AMD), fear of falling, concern about falling, falls, vision impairment

## Abstract

**Purpose:**

To explore the longitudinal impact of central vision loss on concern about falling (CF), over a 12-month period, in people with age-related macular degeneration (AMD).

**Methods:**

Participants included 60 community-dwelling older people (age, 79.7 ± 6.4 years) with central vision impairment due to AMD. Binocular high-contrast visual acuity, contrast sensitivity, and visual fields were assessed at baseline and at 12 months. CF was assessed at both time points using the Falls Efficacy Scale–International (FES-I). Sensorimotor function (sit to stand, knee extension, postural sway, and walking speed) and neuropsychological function (reaction time, symptoms of anxiety and depression) were also assessed at both time points using validated instruments. Falls data were collected using monthly diaries during the 12 months.

**Results:**

CF increased by a small but significant amount over the 12-month follow-up (2.1 units; *P* = 0.01), with increasing prevalence of high levels of CF (FES-I score ≥ 23), from 48% at baseline to 65% at 12 months. Linear mixed models showed that reduced contrast sensitivity was significantly associated with increased concern about falling (*P* *=* 0.004), whereas declines in both visual acuity and contrast sensitivity during the follow-up period were associated with increases in CF over the 12-month follow-up (*P* = 0.041 and *P* = 0.054, respectively), independent of age, gender, falls history, or number of comorbidities.

**Conclusions:**

Higher levels of CF are common in older people with AMD, and levels increase over time; this increase is associated with declines in both visual acuity and contrast sensitivity. These findings highlight the need for regular assessment of both visual acuity and contrast sensitivity to identify those at greatest risk of developing higher CF.

**Translational Relevance:**

Routine assessment of visual acuity and contrast sensitivity in older people with AMD will assist in identifying those at risk of developing high CF.

## Introduction

Age-related macular degeneration (AMD) is the leading cause of blindness in the developed world.^1^ Older people with vision impairment due to AMD experience multiple issues that can negatively affect their health and overall quality of life,^2^ particularly falls and concern about falling (CF).^3^ Falls are the most common cause of injury-related morbidity and mortality in older people.^4^ They are twice as common among older people with AMD^3^^,^^5^^,^^6^ as among those with normal vision, and increased severity of vision loss is associated with higher rates of falls.^3^ Concern about falling is another common issue for older people, with far-reaching implications including activity restriction, reduced quality of life, and, importantly, increased risk of falls.^7^^–^^10^ Previous research has demonstrated a relationship between vision impairment due to AMD and CF with significant associations identified between the level of CF and severity of vision impairment.^11^^,^^12^

Among older people with AMD the rates and levels of vision loss vary, and for many individuals significant declines in visual function are experienced over time.^13^^,^^14^ Levels of CF among this population are also likely to increase over time as vision declines; however, this has not previously been explored. The likelihood of higher levels of CF developing over time may be exacerbated by other risk factors that are strongly linked to CF, which commonly occur within this population, including activity restriction^15^^,^^16^ and depression.^17^^,^^18^ Associations between activity restriction and CF have additional relevance for older people with AMD, given the low levels of activity identified among this population and the association between more severe vision impairment and activity restriction.^19^^–^^21^ Research has also demonstrated that older people with AMD are at higher risk of depression,^22^ and increased severity of vision loss is associated with more severe levels of depression.^3^^,^^23^ It is therefore likely that individuals with deteriorating vision will experience increasing levels of activity avoidance, falls, and depression, which in turn can increase their risk of developing higher levels of CF. However, to date, CF and CF-related activity restriction in AMD have only been investigated in cross-sectional studies.^11^^,^^24^

The aim of this study was to investigate the longitudinal changes in CF using the Falls Efficacy Scale–International (FES-I), which is a validated and widely used instrument, and to explore how changes in visual function and falls over a 12-month period in older persons with AMD are associated with changes in CF. This information would allow a better understanding of CF in older people with AMD, in terms of who is most at risk, and how CF may change with disease progression. A further aim was to explore changes in physical function over the 12 months using a battery of widely used and well-recognized measures.

## Methods

### Participants

Seventy-seven community-dwelling adults over the age of 65 with vision impairment due to AMD were recruited from the low vision clinics at Queensland University of Technology and private ophthalmology practices. Participants were excluded if they had any significant ocular or visual pathway disease, other than normal age-related cataract changes. Other exclusion criteria were: a history of Parkinson's disease, vestibular dizziness or disease, cognitive impairment, or inability to walk household distances without the assistance of another person. The Montreal Cognitive Assessment (MOCA)-Blind version was used to screen for cognitive impairment;^25^ participants scoring less than 14, which reflects moderate to severe cognitive impairment, were excluded.^26^

A total of 60 participants also completed the 12-month follow-up assessment. Participants who did not complete the 12-month follow-up assessment (*n* = 17) were significantly older compared with those who did (mean age 84.8 ± 3.6 years vs. 79.7 ± 6.4 years, respectively; *P* = 0.001), but there was no significant difference in gender distribution (*P* = 0.11). Reasons for withdrawal included poor health, caring for a spouse, moving away, and loss of interest. Binocular visual acuity levels were worse among those who did not attend follow-up, although the difference was not statistically significant: (0.46 ± 0.30 vs. 0.59 ± 0.31, respectively; *P* = 0.18). Baseline FES-I scores did not differ significantly between those who attended the 12-month follow-up and those who withdrew (24.9 ± 11.2 and 24.9 ± 7.5, respectively; *P* = 1.0).

The research followed the tenets of the Declaration of Helsinki, and, prior to participation, informed consent was obtained from all participants after they were given an explanation of the nature and possible consequences of the study. The research was approved by the Queensland University of Technology Human Research Ethics Committee.

### Baseline Demographic and Health History

Demographic information was collected at the baseline visit, including age, gender, years of education, and current medications. Presence of medical comorbidities was determined by self-report, using a list of 13 common medical conditions, including: heart problems, circulation problems, high or low blood pressure, neurological issues, diabetes, arthritis, osteoporosis, chronic pulmonary problems, problems relating to digestion, kidney problems, hearing impairment, and speech impairment, based on categorizations used in previous studies.^27^ Participants were also asked to report the use of any walking aids.

### Vision Assessment

At the baseline and follow-up study visits, participants underwent an eye examination, which included slit-lamp assessment and grading of lens opacities using the Lens Opacities Classification System, Version III,^28^ as well as retinal examination using indirect ophthalmoscopy. Severity of AMD was graded according to the Beckman classification scale by an experienced optometrist (UW), based on drusen size, pigmentary abnormalities, and presence/absence of geographic atrophy or neovascular AMD.^29^ The prescription and type of habitual spectacle correction (e.g., single vision, multifocal, progressive) were also recorded.

Visual acuity was assessed binocularly using an Early Treatment for Diabetic Retinopathy Study logMAR chart (Good-Lite Company, Elgin, IL) at a working distance of 3 m and luminance of 130 cd/m^2^. Participants completed the assessment wearing their habitual distance correction. Testing distance was reduced to 1 m if a measure of visual acuity could not be obtained at 3 m. Participants were encouraged to guess letters, with a termination rule of four or more letters reported incorrectly; results were recorded using letter-by-letter scoring.

Contrast sensitivity was measured binocularly using the photographic printed version of the Melbourne Edge Test^30^ (National Vision Research Institute, Melbourne, Australia), administered at a working distance of 40 cm, with an ambient room illumination of 440 lux on the chart and a +2.50D near correction worn over any habitual distance prescription. Participants were instructed to report the orientation of the edge within each circular patch until two consecutive orientations were identified incorrectly. The last correctly identified orientation was recorded as the contrast sensitivity threshold, measured in decibel units.

Visual fields were assessed binocularly as described in previous research,^31^ using the 30-2 program with the SITA Fast strategy on a Humphrey Field Analyzer Model 750 (Carl Zeiss Meditec, Jena, Germany). This approach of measuring visual fields during binocular viewing minimizes errors that may be incurred through integration of monocular visual fields, particularly for participants who adopt monocular eccentric fixation. In brief, participants wore their habitual distance prescription with a +3.00D working distance correction, using full aperture lenses in a half-eye trial frame, and fixation was monitored by the examiner through the video monitor.

### Concern About Falling

Concern about falling was assessed using the FES-I,^32^ which assesses CF across 16 common daily living activities, such as getting dressed, shopping, and using stairs, based on a four-point scale (1 = not at all concerned to 4 = very concerned). The score is determined by summing all responses, with higher scores indicating higher levels of CF (range, 16–64). A score of ≥23 was classified as a high level of CF, and a score of ≥28 was considered a very high level of CF, based on the approach used in a previous study of community-dwelling older people.^33^ The FES-I has excellent psychometric properties and internal reliability (Cronbach's alpha value of 0.96^32^), with high test–retest reliability (0.96) and validity.^32^

### Sensorimotor Assessment

Functional lower limb performance was assessed using the five-times sit to stand test.^34^ Participants began in a seated position and were instructed to rise as quickly as possible to standing, without the aid of their arms. Time taken to stand up and sit down five times was measured in seconds, as previously described.^35^ Lower limb strength was assessed by measuring the isometric quadriceps strength (knee extensor muscles) in the dominant leg, using a spring gauge dynamometer. Participants were seated with the hip and knee joints positioned at 90° and the spring gauge strap attached above the ankle. Participants were asked to push their dominant lower leg out as far as they were able, and a strength measurement (in kg force) was obtained; a mean value was calculated from three measurements.^36^

Postural sway was measured while the participant stood with eyes open on a foam surface, based on a standardized protocol.^37^ Participants were positioned on a force platform (Hur Labs, Tampere, Finland), and instructed to stand as still as possible for a period of 30 seconds. The center of pressure trace length was measured in millimeters. Participants wore their habitual distance correction and were instructed to look straight ahead at a high-contrast 20 × 30-cm distance fixation target during the test. Each participant's self-selected walking speed (m/s) was measured along a 23-m well-lit indoor walkway with no obstructions. Participants were instructed to walk at their usual speed to the far end of the corridor, touch the wall at the end, and immediately return to the starting point. Time taken was recorded in seconds using a stopwatch.

### Neuropsychological Assessment

Cognitive status was assessed using the MOCA-Blind. This measure is widely used as a screening tool in persons with vision impairment, and the maximum possible score is 22.^26^ Simple hand reaction time was assessed using a hand-held electronic timer in the form of a modified computer mouse.^36^ Participants were seated during the test and asked to press the button on the mouse as soon as they observed the appearance of a bright light-emitting diode. After five practice tests, 10 measurements were recorded, and an average score was calculated in seconds. Depressive symptoms were evaluated using the Patient Health Questionnaire-9, a validated measure used widely in previous research, including among populations with vision impairment;^38^ higher scores indicate more severe depression.^39^ Symptoms of anxiety were assessed using the Geriatric Anxiety Inventory, a well-validated measure designed specifically for use in older people; higher scores indicate higher levels of anxiety.^40^

### Prospective Falls

Falls were recorded prospectively over the 12-month follow-up using monthly falls diaries.^41^ Participants were asked to record any falls each day, to provide details of any fall-related injuries, and to return the diaries at the end of each month via mail. A definition of a fall was included in each diary: *A fall is where you came to rest unintentionally on the ground or any other lower level.* If a diary was not returned within 3 weeks, the participant was reminded by means of a phone call.

### Statistical Analysis

Statistical analyses were performed using SPSS Statistics 25 (IBM, Armonk, NY) and statistical software R 4.0.5 (R Foundation for Statistical Computing, Vienna, Austria) using the “lme4” package, and the level of significance was set at *P* < 0.05. Descriptive statistics were used for demographic, CF, vision, sensorimotor, and neuropsychological variables at baseline and at 12-month follow-up; mean and standard deviation and median and interquartile range were used for normal and non-normal distributions, respectively. For continuously distributed variables, changes over the 12-month follow-up period were assessed using paired *t*-tests; bootstrapping was used where the sampling distribution of variables was non-normally distributed, and categorical variables were analyzed using McNemar's test.

Linear mixed models were used to explore the longitudinal associations between vision function and CF, as they account for clustering of observations within individuals, allow for the inclusion of time-invariant and time-varying predictors, and flexibly accommodate for missing data.^42^ For the time-varying vision predictors, two variables were created to disaggregate the between-person and within-person components, an approach used previously in longitudinal studies.^43^ To obtain within-person effects, the vision variables were person mean-centered, reflecting the degree to which an individual's momentary vision measure differs from their average level. To obtain between-person effects, variables were averaged over the two time points and grand mean-centered, reflecting the degree to which an individual's average level of a variable differs from the total sample mean.

The first set of models explored the association between each visual measure (visual acuity and contrast sensitivity) and CF in separate models, which included both the within- and between-person components of the vision measure to test their relative contributions, and a time variable (baseline and 12-month follow up). These models were further adjusted for time-invariant covariates based on their assumed confounding effect and included age (at baseline), gender, and number of comorbidities (at baseline).

Finally, exploratory models were run to explore the combined associations of all vision measures, including both within- and between-person components, with CF adjusted for age, gender, and number of comorbidities. Falls are a potential mediating factor between vision and CF, being on the casual pathway, whereby reduced vision increases the risk of falls, which may lead to increased CF. The final exploratory model included the number of prospective falls reported during the 12-month follow-up period as a covariate, to explore how this influenced the association between vision measures and CF. Given the likely correlations between visual acuity and contrast sensitivity, multicollinearity was checked in these models by calculating the variance inflation factor.

## Results

The mean age of the 60 participants was 79.7 years (SD = 6.4), and 42 were female (70%). The mean number of years of education was 12.7 (SD = 5.2). Participants reported a mean of 4.0 (SD = 1.7) comorbidities; the most frequent conditions were arthritis (70%), hearing loss (58%), and high blood pressure (55%). Thirty-five participants reported taking four or more regular prescription medications (58%), and 23 regularly used a mobility aid (38%). Most participants were graded as having intermediate or late AMD based on the Beckman classification (Table 1); binocular visual acuity levels indicated mild to moderate levels of vision impairment (mean, 0.46 logMAR; SD = 0.3; range, 0.00–1.22). Over half of the group had undergone surgery for cataract; 37 were pseudophakic in their better eye (62%), and the remainder had mild lens changes in their better eye. The most common forms of spectacle correction used habitually were bifocals (*n* = 21; 34%), single-vision distance (*n* = 15; 25%), progressives (*n* = 14; 23%), and single-vision reading (*n* = 7; 11%).

**Table 1.** AMD Grading (Beckman Classification) at Baseline Visit (*n* = 60)

Beckman Classification	Better Eye, *n* (%)	Worse Eye, *n* (%)
No/normal aging changes	4 (7)	0
Early AMD	3 (5)	0
Intermediate AMD	14 (23)	6 (10)
Late AMD	37 (62)	52 (87)
Missing^a^	2 (3)	2 (3)
Total	60 (100)	60 (100)

a Data were not obtained for two participants whose physical status prohibited examination with the slit lamp. Both had longstanding diagnoses of bilateral AMD from their treating ophthalmologist, with significant bilateral vision loss and binocular visual acuities worse than 0.30 logMAR.

Table 2 shows values at baseline and 12-month follow-up for FES-I scores, as well as the vision, sensorimotor, and neuropsychological measures; prospective falls recorded over the 12-month follow-up are also reported in the table. Over the 12-month follow-up, levels of CF increased significantly, with a 2.1-unit increase in FES-I scores (*P* = 0.011). The proportion of participants with high concern (FES-I score ≥ 23), also increased significantly between baseline and 12-month follow-up (*P* = 0.021), and the increase in the proportion of participants with very high concern (FES-I score ≥ 28) was even more significant (*P* ≤ 0.001). The majority of participants who reported high concern at baseline also reported high concern at follow-up; 29 participants reported high CF (FES-I ≥ 23) at baseline, and 26 of these remained high CF at follow-up. An additional 13 participants who had reported low levels of CF at baseline had transitioned to high CF at the 12-month follow-up.

**Table 2.** Values for Concern about Falling; Vision, Sensorimotor, and Neuropsychological Function; and Prospective Falls for Participants with AMD at Baseline and 12-Month Follow-Up (*n* = 60)

Measure	Baseline	Follow-up	*P*
Concern about falling			
FES-I, mean (SD)	24.9 (7.5)	27.0 (8.5)	**0.011**
High concern (FES-I ≥ 23), *n* (%)	29 (48)	39 (65)	**0.021**
Very high concern (FES-I ≥ 28), *n* (%)	21 (35)	25 (42)	**≤0.001**
Vision, mean (SD)			
Binocular visual acuity (logMAR)	0.46 (0.30)	0.52 (0.34)	**0.008**
Contrast sensitivity (Melbourne Edge Test) (dB)	17.6 (4.8)	16.8 (5.2)	**0.014**
Visual field 30-2 mean deviation (dB)	−3.30 (3.83)	−3.54 (4.42)	0.13
Sensorimotor, mean (SD)			
Sit to stand (s)	20.3 (13.7)	21.7 (14.6)	0.20
Knee extension strength (kg)	20.0 (8.7)	18.6 (7.9)	**0.028**
Postural sway on foam: trace length (mm)	967 (401)	1049 (394)	0.13
Walking speed (m/s)	1.05 (0.26)	1.00 (0.26)	**0.004**
Neuropsychological			
MOCA–Blind, mean (SD)	19.9 (1.8)	19.9 (2.1)	0.89
Reaction time (s), mean (SD)	0.34 (0.15)	0.41 (0.29)	**0.049** ^a^
Geriatric Anxiety Inventory,^b^ median (IQR)	1.0 (0.0–4.0)	1.0 (0.0–3.8)	0.38
Patient Health Questionnaire-9,^c^ median (IQR)	3.0 (0.0–5.0)	2.0 (0.0–4.0)	0.16
Prospective falls ^d^			
Falls, mean (SD)	—	1 (1.5)	—
Range, *n*	—	0–7	—
Participants experiencing no falls, *n* (%)	—	24 (42)	—
Participants experiencing one or more falls, *n* (%)	—	33 (58)	—
Participants experiencing two or more falls, *n* (%)	—	15 (26)	—
Participants experiencing one or more injurious falls, *n* (%)	—	20 (35)	—

Significant *P* values are highlighted in bold (paired samples *t*-test for continuous variables, McNemar's test for categorical variables).

a Values confirmed with robust methods (bootstrapping).

b Possible score range 0 to 20; higher scores indicate more symptoms of anxiety.

c Possible score range 0 to 27; higher scores indicate more symptoms of depression.

d Values are for 57 participants who returned completed falls diaries over the 12-month follow-up.

An overall reduction in vision was observed between baseline and the 12-month follow-up, with significant declines in visual acuity (0.06 logMAR, or a reduction of three letters; *P* = 0.008) and contrast sensitivity (0.8 log units; *P* = 0.014). Twenty-seven participants were undergoing anti-vascular endothelial growth factor therapy (anti-VEGF). Changes in visual acuity for these participants ranged from –0.36 to +0.34 logMAR, with 13 participants showing one or more lines worsening of visual acuity, five showing one or more lines of improvement in visual acuity, and nine showing minimal change in visual acuity (less than one line change). Of these 27 participants, changes in contrast sensitivity ranged from –8 dB to +4 dB, with four participants showing a worsening of ≥3 dB, one showing an improvement of ≥3 dB, and 22 showing minimal change in contrast sensitivity (<3 dB).

Declines in physical function were also found, with walking speed and knee extension strength worsening significantly over the 12 months (*P* = 0.004 and *P* = 0.028, respectively). No significant changes were found for levels of depression and anxiety (*P* = 0.16 and *P* = 0.38, respectively).

### Prospective Falls Over 12 Months

Prospective falls data was collected over the 12-month follow-up for 57 of the 60 participants (three participants did not return the monthly falls diaries); see Table 2. There were no significant differences in age between fallers (mean age, 80.9 years; SD = 6.1) and non-fallers (mean age, 78.8 years; SD = 6.8) (*P* = 0.20). The proportion of participants who regularly wore multifocal lenses (including bifocals and progressive lenses) did not differ significantly between fallers and non-fallers (53% and 70%, respectively; rate ratio, 1.11; 95% confidence interval [CI], 0.53–2.32). The mean baseline FES-I scores were not significantly different between fallers and non-fallers (25.2, SD = 6.9 vs. 23.2, SD = 7.8, respectively; *P* = 0.30).

### Visual Associations with Changes to FES-I

Associations between vision measures and CF over 12 months were assessed for visual acuity and contrast sensitivity, as these measures changed significantly over the 12-month follow-up. Table 3 presents the parameter estimates from the linear mixed models exploring the associations between each vision measure and CF in separate models, including the between- and within-person components. For the visual acuity models, there was no significant between-person effect of visual acuity (*P* > 0.13), whereas the within-person effects were significant (*P* = 0.04), with a 0.93-unit (95% CI, 0.07–1.79) increase in FES-I scores for each one-line reduction in visual acuity over the 12-month follow-up period. For the contrast sensitivity models, there was a significant between-person effect of contrast sensitivity (*P* = 0.002), with a 0.56-unit (95% CI, 0.23–0.89) increase in FES-I scores for each 1-dB reduction in contrast sensitivity (Model 2). The within-person effects of contrast sensitivity were close to significant (*P* = 0.053), with a 0.61-unit (95% CI, 0.002–1.22) increase in FES-I scores for each 1-dB reduction in contrast sensitivity over the 12-month follow-up period. In all models, there was a significant increase in FES-I scores over time (*P* < 0.043), and higher numbers of comorbidities were strongly associated with higher FES-I scores (*P* = 0.001).

**Table 3.** Parameter Estimates for the Linear Mixed Models for the Associations of Visual Acuity and Contrast Sensitivity with Concern About Falling

	Model 1			Model 2		
	Estimate	SE	*P*	Estimate	SE	*P*
Visual acuity						
Time (ref: baseline)	**1.75**	**0.84**	**0.043**	**1.75**	**0.84**	**0.043**
Between-person (per 1-line reduction)	0.48	0.31	0.13	0.38	0.29	0.19
Within-person (per 1-line reduction)	**0.93**	**0.44**	**0.040**	**0.93**	**0.44**	**0.040**
Age (per year)	—	—	—	0.18	0.14	0.20
Gender (ref: male)	—	—	—	**3.82**	**1.74**	**0.032**
Number of comorbidities	—	—	—	**1.80**	**0.52**	**0.001**
Contrast sensitivity						
Time (ref: baseline)	**1.83**	**0.84**	**0.033**	**1.83**	**0.84**	**0.033**
Between-person (per 1-dB reduction)	**0.74**	**0.17**	**<0.001**	**0.56**	**0.17**	**0.002**
Within-person (per 1-dB reduction)	0.61	0.31	0.053	0.61	0.31	0.053
Age (per year)	—	—	—	0.05	0.13	0.71
Gender (ref: male)	—	—	—	2.72	1.65	0.10
Number of comorbidities	—	—	—	**1.70**	**0.48**	**0.001**

Bold values indicate a statistically significant result. Higher FES-I scores indicate greater concern about falling.

Table 4 presents the parameter estimates from the linear mixed models exploring the association between both vision measures and CF, including the between- and within-person components. Variance inflation values were all below 2.2, indicating no multicollinearity concerns. In Model 1, reduced contrast sensitivity (between-person effect) was significantly associated with greater FES-I scores (*P* = 0.004), yet there was no association with reduced visual acuity (between-persons effect). Furthermore, there were significant effects of within-person changes in vision, particularly within-person reductions in visual acuity that were strongly associated with higher FES-I scores (*P* = 0.04), and there was an almost significant association between within-person reduction in contrast sensitivity and higher FES-I scores (*P* = 0.054). When the number of prospective falls during the 12-month follow-up period was included as a potential mediator (Model 2), the effect of visual acuity and contrast sensitivity (between-person components) on CF remained largely unchanged; thus, falls are not likely to be a significant mediator between changes in vision and CF. The number of prospective falls was not a significant predictor of FES-I scores (*P* = 0.50) in this model.

**Table 4.** Linear Mixed Models for the Effects of Visual Acuity and Contrast Sensitivity on CF

	Model 1			Model 2		
	Estimate	SE	*p*-value	Estimate	SE	*p*-value
Time (ref: baseline)	1.26	0.85	0.15	1.26	0.85	0.15
Visual acuity, between-person (per 1-line reduction)	−0.19	3.28	0.56	−0.16	3.29	0.62
Visual acuity, within-person (per 1-line reduction)	**0.9**	**4.29**	**0.041**	**0.9**	**4.29**	**0.041**
Contrast sensitivity, between-person (per 1-dB reduction)	**0.63**	**0.21**	**0.004**	**0.64**	**0.21**	**0.004**
Contrast sensitivity, within-person (per 1-dB reduction)	0.58	0.3	0.054	0.58	0.3	0.054
Age (per year)	0.06	0.13	0.64	0.06	0.13	0.65
Gender (ref: male)	2.56	1.67	0.13	2.48	1.67	0.14
Number of comorbidities	**1.65**	**0.48**	**0.001**	**1.64**	**0.48**	**0.001**
Number of falls	—	—	—	0.33	0.49	0.50

Bold values indicate a statistically significant result. Higher FES-I scores indicate greater concern about falling.

## Discussion

In this study, significant increases in CF were found over a 12-month period for community-dwelling older people with AMD. Importantly, adults with reduced contrast sensitivity were more likely to express high levels of CF, and, over time, reductions in both visual acuity and contrast sensitivity at an individual level were associated with increases in CF. People with a higher number of comorbidities are at higher risk of developing high CF, but falls history does not predict CF level. These findings highlight the short timeframe over which significant increases in CF can occur for older people with AMD, and its relationship with changes in vision function.

Levels of CF were high in the current study, when compared with previous research. The highest levels of CF (defined as scores ≥ 28) were reported by 35% and 42% of study participants at baseline and follow-up, respectively, as compared with 22% reported in a study involving older women with a history of falls, a population at risk of CF due to their gender and falls history.^44^ In the current study, high CF (FES-I ≥ 23) was identified in just under half of the participants (48%) at baseline, and the proportion increased significantly to 65% at follow-up, with a significant mean FES-I score increase of 2.1 units over the 12-month period. This increase is twice that found previously in a general population of 463 community-dwelling older people over a 12-month period (1-unit mean change).^33^ Fourteen participants (23%) transitioned from a lower to a higher concern category (10 from low to high CF and a further four from high to very high CF), suggesting that, as a group, the increase in levels of CF was of clinical significance. In most individuals, high levels of CF persisted over time, with 90% of those who reported high CF (FES-I ≥ 23) at baseline also reporting high CF at follow-up. This proportion is higher than the levels of persistent CF (45%–80%) reported in general populations of older people.^8^^,^^45^^,^^46^ Although fluctuations in CF are likely to occur in response to day-to-day experiences, risk factors have been identified for persistent CF in general populations, including female gender and a history of falls.^46^ The current findings suggest that older people with AMD are more likely to experience high levels of CF and that declines in vision are associated with persistent CF.

The proportion of participants experiencing single or multiple falls (57% and 26%, respectively) during the 12-month follow-up was also high but consistent with rates reported in previous studies of visually impaired populations.^3^^,^^47^^–^^49^ Importantly, these fall rates are high when compared with those reported in community studies (around 30% and 16% for single and multiple falls, respectively),^48^^,^^49^ highlighting the need to better understand and address risk factors for falls in this population. Injurious falls were also common, with 35% of the participants reporting that they had incurred injuries during a fall and 25% requiring medical attention.

No association was found between change to FES-I score over the 12-month follow-up and the number of prospective falls that occurred during this period. Indeed, the relationship between falls and CF is complex, with CF being influenced by many other factors, including mobility and balance, as reported in studies of general populations.^50^ More research is required to better understand the causal relationship between falls and CF across all populations. Importantly, the lack of a clear directional association between CF and falls illustrates that, for older people with vision loss due to AMD, CF is a problem in its own right and not simply a consequence of experiencing a fall.

There was a mean decline in visual acuity and contrast sensitivity over the 12-month follow-up, which is not surprising given the progressive nature of AMD and the limitations of current treatments. Even among participants treated with anti-VEGF whose visual acuity improved, there were still reductions in contrast sensitivity, suggesting that aspects of functional vision did not improve with these treatments. Previous research has demonstrated that people with AMD can have significant contrast sensitivity deficits while their visual acuity remains relatively normal.^51^ Although there is some evidence to suggest that treatment with anti-VEGF therapy can initially result in improvements to contrast sensitivity even when visual acuity remains stable, the effects of anti-VEGF therapy on contrast sensitivity in the longer term remain largely unstudied.^52^

Importantly, although reduced contrast sensitivity best predicted higher levels of CF, declines in both visual acuity and contrast sensitivity were associated with increases in CF over the 12-month follow-up, independent of falls history and number of comorbidities. Associations between CF and visual function found in this longitudinal study are in line with previous cross-sectional research of older people with AMD, which have identified significant between-person associations for reduced contrast sensitivity with increased levels of CF and CF-related activity restriction and weaker associations with visual acuity.^11^^,^^24^ Previous research has also identified that contrast sensitivity is the best predictor of physical performance with respect to falls and balance.^3^^,^^53^^,^^54^ These findings collectively highlight the importance of regularly assessing both visual acuity and contrast sensitivity in low-vision rehabilitation settings, in order to best identify those individuals at the highest risk of both experiencing falls and developing CF over time as their vision declines. The findings can also be used to inform the design of future vision rehabilitation programs incorporating interventions to address excessive levels of CF.

Declines in physical function were also observed between baseline and 12-month follow-up, with significantly slower walking speeds and poorer knee extensor strength at 12-month follow-up, when compared with baseline values. Previous research has identified that older people with vision impairment have significantly poorer physical function when compared with their normally sighted peers.^55^^–^^57^ The current study is the first to examine physical declines among older people with AMD over time; declines are likely to be in part secondary to activity restriction associated with vision impairment, given the high levels of activity restriction reported in previous studies of older people with AMD.^20^^,^^21^

There were no significant changes in the level of depression and anxiety symptoms between baseline and the 12-month follow-up. It is possible that symptoms of depression increase over time in this population and that the non-significant findings in the current study are due to under-representation of those individuals with more severe levels of vision loss and activity restriction; a larger longitudinal study is required to confirm this.

Strengths of this study include the novel longitudinal design for investigating CF over time in an AMD population; the comprehensive assessment of CF, vision, sensorimotor, and neuropsychological function using widely recognized, validated measures; and the use of prospective falls diaries. Limitations include the small sample size and under-representation of older people with more severe vision loss. Future studies with larger sample sizes are recommended to improve the generalizability of these findings. Additionally, incorporating validated measures of activity levels, such as accelerometers, would improve our understanding of the associations among CF, physiological condition, and activity levels for older people with AMD and assist in the design of interventions to reduce falls and manage CF in this population.

## Conclusions

In this study, which, to the best of our knowledge, is the first to explore the longitudinal relationship between changes in visual function and CF in older people with AMD, significant declines in vision and physical function, and increased levels of CF occurred over a 12-month period among older people with vision impairment due to AMD. Importantly, increased CF was associated with reduced visual acuity and contrast sensitivity. This highlights the need to regularly assess both measures of vision function for patients with AMD when attending vision rehabilitation settings to identify those at greatest risk of developing higher CF. Individuals identified as high risk for CF could then be directed to interventions to manage the physical risk factors for falls and CF that are tailored to the specific needs of older people with vision impairment.
